# Regulation of Tumor Immunity by Lysophosphatidic Acid

**DOI:** 10.3390/cancers12051202

**Published:** 2020-05-10

**Authors:** Sue Chin Lee, Mélanie A. Dacheux, Derek D. Norman, Louisa Balázs, Raul M. Torres, Corinne E. Augelli-Szafran, Gábor J. Tigyi

**Affiliations:** 1 Department of Physiology, University of Tennessee Health Science Center, Memphis, TN 38163, USA; slee84@uthsc.edu (S.C.L.); mdacheux@uthsc.edu (M.A.D.); dnorman7@uthsc.edu (D.D.N.); 2 Department of Pathology and Laboratory Medicine, University of Tennessee Health Science Center, Memphis, TN 38163, USA; cziczam@comcast.net; 3 Department of Immunology & Microbiology, University of Colorado School of Medicine, Denver, CO 80045, USA; raul.torres@cuanschutz.edu; 4 Department of Chemistry, Drug Discovery Division, Southern Research, Birmingham, AL 35205, USA; caugelli-szafran@southernresearch.org

**Keywords:** immunosurveillance, immunoediting, immunosuppression, lysophosphatidic acid, LPA, autotaxin, cytotoxic T cells, immune checkpoint, immune cells, tumor-associated macrophages, tumor microenvironment, LPAR5

## Abstract

The tumor microenvironment (TME) may be best conceptualized as an ecosystem comprised of cancer cells interacting with a multitude of stromal components such as the extracellular matrix (ECM), blood and lymphatic networks, fibroblasts, adipocytes, and cells of the immune system. At the center of this crosstalk between cancer cells and their TME is the bioactive lipid lysophosphatidic acid (LPA). High levels of LPA and the enzyme generating it, termed autotaxin (ATX), are present in many cancers. It is also well documented that LPA drives tumor progression by promoting angiogenesis, proliferation, survival, invasion and metastasis. One of the hallmarks of cancer is the ability to modulate and escape immune detection and eradication. Despite the profound role of LPA in regulating immune functions and inflammation, its role in the context of tumor immunity has not received much attention until recently where emerging studies highlight that this signaling axis may be a means that cancer cells adopt to evade immune detection and eradication. The present review aims to look at the immunomodulatory actions of LPA in baseline immunity to provide a broad understanding of the subject with a special emphasis on LPA and cancer immunity, highlighting the latest progress in this area of research.

## 1. Introduction

Lysophosphatidic acid (LPA) is a bioactive lipid involved in regulating a wide range of biological responses in neoplastic cells as well as non-transformed cells. The effects of LPA on malignant transformation, migration, metastasis, invasion, proliferation, survival, angiogenesis, and therapy resistance have been reviewed in detail in [1,2,3,4,5,6] and will not be discussed here. LPA elicits its physiological and pathological effects via cognate extracellular or intracellular receptors expressed in almost every cell type and tissue. These receptors include the extracellular G-protein coupled receptors (GPCRs) LPAR1-6 and the intracellular peroxisome proliferator-activated receptor gamma (PPARγ). It is well documented that LPA is secreted by various cell types such as platelets, adipocytes, fibroblasts, as well as by cancerous cells. In fact, the lysophospholipase D enzyme responsible for the major production of LPA in biological fluids, termed autotaxin (ATX) and known as ectonucleotide pyrophosphatase/phosphodiesterase 2 (ENPP2), was first identified in melanoma cells [7]. There are numerous publications on the upregulation of ATX-LPA signaling axis in many types of cancers, suggesting that this is a common pathway that cancer hijacks for its progression [2,4,8]. In particular, high levels of LPA and ATX were found to be present in the ascites of ovarian cancer patients [9,10,11,12] and are associated with a short relapse-free survival [12]. Likewise, other cancers such as hepatocellular carcinoma (HCC) [13,14], pancreatic cancer [15], and follicular lymphoma [16] all reported increased levels of LPA and/or ATX. Moreover, studies have found that in the mammary tumor microenvironment (TME), breast cancer cells, via secretion of tumor-derived cytokines, reprogram adjacent adipocytes to increase secretion of ATX, which further drive tumor growth, metastasis, and therapy resistance [17,18,19,20]. However, the role of LPA in the regulation of immunity has received less attention than in the physiology and pathophysiology of other organ systems. The reasons behind this selective neglect may have stemmed from some early reports that used Jurkat T cells and human peripheral blood neutrophils to test for LPA-activated Ca^2+^ responses only to find lack of responsiveness [21]. This was in sharp contrast to fibroblasts that showed robust Ca^2+^ mobilization following LPA exposure. Another research group also used a Jurkat cell line and found that LPA activated robust Ca^2+^ transients [22]. These authors found that LPA desensitized the Ca^2+^ response to subsequent application of sphingosylphosphorylcholine (SPC) or lysophosphatidylserine. Interestingly, in their experiments LPA did not seem to reduce the Ca^2+^ signal elicited by activation of the T cell receptor (TCR). LPA in this Jurkat cell line served as a mitogen in the presence of low serum concentration and increased IL-2 secretion by five-fold after cotreatment with phorbol myristate acetate (PMA) compared to PMA alone [22]. We found that cross-desensitization between LPA and SPC response is likely due to 1-O-cis-alk-1′-enyl-2-lyso-sn-glycero-3-phosphate contamination of the SIGMA—Aldrich brand SPC [23]. It was only years later that LPA responsiveness was demonstrated in T lymphocytes [24], B cells [25,26,27], eosinophils [28], neutrophils [29], macrophages [30], mast cells [31], dendritic cells (DC) [32,33,34], and natural killer (NK) cells [35,36,37]. Although these studies clearly and firmly established the immunoregulatory role of LPA in various immune cell types, most of them were conducted prior to the identification of all currently known LPA receptors (LPAR1-6), and not specifically in the context of cancer immunity.

**Figure 1:**
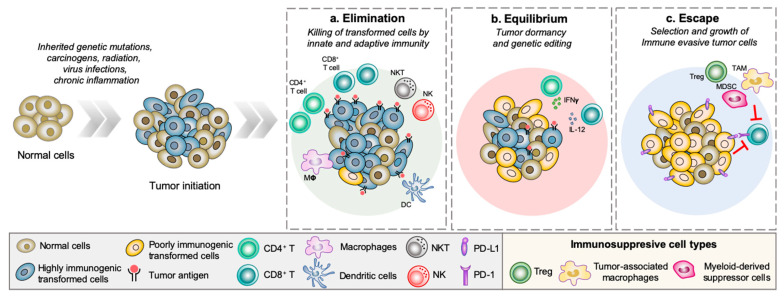
Cancer immunoediting concept–the dual host-protecting and tumor-sculpting actions of the immune system proposed by Dunn et al. [24]. Oncogenic stimuli cause normal cells to undergo transformation and become tumorigenic. Transformed cells are recognized and eradicated by the innate and adaptive immune systems during the elimination phase. In the equilibrium phase, surviving tumor cells continue to adapt to the constant anti-tumor immune pressure. The escape phase is largely associated with the outgrowth of immunoedited tumor cells capable of evading the immune systems through various means; loss of immunogenicity, loss of antigenicity, and/or orchestrating an immunosuppressive microenvironment (i.e., recruitment of immunosuppressive cell types such as regulatory T cells (Treg), tumor-associated macrophages (TAM), and myeloid-derived suppressor cells (MDSC)).

Cancer immunity is a complex and dynamic process best explained using the cancer immunoediting theory proposed by Dunn et al. in 2002. This theory divides cancer immunity into three phases: elimination, equilibrium, and escape (Figure 1). In the first phase, the innate and adaptive immune system detects and eliminates nascently transformed cells (i.e., cancer immunosurveillance). Tumor cells that manage to survive the anti-tumor immune response enter into an equilibrium phase—a period of low tumor growth rate as the surviving tumor cells continue to mutate and adapt to the constant anti-tumor immune pressure (i.e., editing). Tumor subclones that successfully develop immune tolerant mechanisms escape detection and eradication by the immune system, and enter an unrestrained growth period, also known as the escape phase. The cancer immunoediting concept provided an important insight into how tumor cells are shaped and sculpted by the continuous interaction with immune cells of the TME and vice versa [38]. In order to escape immune-mediated detection and eradication, tumor cells must develop strategies that disrupt the proper execution of the anti-tumor immune response. These strategies include shutting down T cell activation by means of binding to immune checkpoint receptors such as program death protein 1 (PD-1) or cytotoxic T-lymphocyte-associated protein 4 (CTLA-4), impairing antigen presentation machinery, secretion of immunosuppressive factors, and recruitment of immunosuppressive cell types into the TME [39].

Cellular communication inside the TME is led by a complex system composed by cytokines, growth factors, enzymes, and lipids. Recent studies have provided some insights that altered lysophospholipid profile such as LPA in the TME may be one of the strategies that tumor cells utilize to escape anti-cancer immune response [12,40,41]. This is not surprising based on the numerous reports mentioned above that established an immunoregulatory role for LPA. The role of endogenous LPA in possibly limiting tumor immunity was demonstrated recently using ATX heterozygous mice (*Enpp2*^+/−^) that have 50% lower circulating LPA level. Briefly, C57BL/6 wild type or *Enpp2^+/^*^−^ mice were treated with a cocktail of anti-CD40, pI:C and OVA, an immunization protocol known to increase the number of endogenous OVA-reactive CD8^+^ T cells [42]. The cytotoxic function of CD8^+^ T cells in vivo was measured by transferring CD45.1 C57BL/6 cells pulsed with the SIINFEKL peptide (which serve as ‘target cells’) into immunized wild type C57BL/6 or *Enpp2^+/^*^−^ mice. Quantification of the number of antigen-specific peptide pulsed target cells revealed that the in vivo CD8^+^ T cell cytotoxicity was significantly higher in *Enpp2*^+/-^ mice (2-fold increase in cell killing) compared to wild type C57BL/6 mice, suggesting that systemic levels of LPA can modulate anti-tumor immune response [43]. Findings from a recent study aimed at understanding the interrelation between sphingolipid, LPA signaling axis, and immune-related genes in the context of ovarian cancer found that S1P and one of its receptors S1PR4, along with the ATX/LPA/LPAR5-6 signaling axes were critical for immune infiltrates. Surprisingly, increased levels of enzymes associated with S1P generation, such as sphingosine kinase, and LPA production, such as ATX, were detected in immune high tumors (i.e., high expression of the immune-associated markers CD163, CD14, CD68, CD45, CD20, and CD3E). In immune low tumors, an increased expression of ceramide kinase and PPAP2C (the gene encoding lipid phosphate 2, LPP2, which is responsible for the breakdown of LPA) were detected. Additionally, LPAR2 and LPAR3 were also enhanced in immune low tumors [40]. However, the exact regulatory roles played by each member of the sphingolipid/lysophosphatidate signaling axis in the context of tumor-immune interactions remains to be elucidated. These lines of evidence coupled with the roles of LPA in regulating immune cell functions raise the question of whether high levels of LPA within the TME may serve as an inhibitory mechanism that regulates cancer immunity. In this context, the surface expression of functional LPAR subtypes is a critical unanswered question in order to predict the effects of LPA on the tumor cells, lymphoid cells, and cell types of the TME. An equally pressing question is the cellular origins of ATX in the TME. These questions should take center stage of future investigations.

Studies that investigated the immunomodulatory functions of LPA in the context of cancer immunity were for the most part underappreciated but have begun to gain more interest recently. In the present review, we summarize some of the earlier studies that led to the discovery of the immunomodulatory functions of LPA in various immune cell types of innate and adaptative immunity with a special emphasis on reports that investigated the immunoregulatory functions of LPA in the context of cancer immunity.

## 2. ATX-LPA-LPAR Signaling Axis in Regulating Innate Immunity

Part of the cancer immunosurveillance process involves the deployment of polymorphonuclear leukocytes (including neutrophils, eosinophils, and basophils), mast cells, monocytes, macrophages, DC, and NK cells. The activation of these innate immune cells will in turn, trigger adaptative immunity, recruiting new actors such as T and B cells. It is important to point out that in the field of oncology, current knowledge in the role of LPA on cells participating in innate immunity is limited to macrophages and NK cells. In fact, how LPA regulates polymorphonuclear leukocytes, mast cells and DC, in the context of cancer, has not been investigated yet. However, some studies have highlighted interesting features of LPA in other pathological contexts. In this section, we discuss both the current knowledge and the lack thereof on how LPA regulates cells of the innate immunity.

### 2.1. LPA in the Regulation of Macrophage and Tumor Associated Macrophage

Among the cells that participate in innate immunity, macrophages are the most studied. Macrophages can arise from embryonic progenitor cells, adult hematopoietic stem cell progenitor cells, or monocyte-derived cells. Interestingly, in both humans and mice, LPA has been reported to convert monocytes into macrophages via activation of PPARγ [44]. PPARγ is an important transcription factor that regulates immune functions such as the inhibition of macrophage proinflammatory genes [45,46] and lung immunity [47]. In breast cancer, LPAR3 expression was associated with macrophage infiltration [48]. Whereas in an experimental model of colitis-associated cancer, it was demonstrated that *Lpar2*^−*/*−^ mice presented reduced macrophage infiltration and less tumors than the wild type or *Lpar2^+/^*^−^ mice [49]. On the contrary, in colorectal cancer (CRC), silencing 1-acylglycerol-3-phosphate O-acyltransferase 4 (Agpat4) induced the production of LPA from CRC cells and consequently polarized peritoneal macrophages in a M1 phenotype through LPAR1 and LPAR3. The authors demonstrated that LPA stimulated the p38 mitogen-activated protein kinase (MAPK) and the p65 subunit of nuclear factor kappa-light-chain-enhancer of activated B cells (NF-κB) signaling pathways in M1 macrophages leading to an increase in proinflammatory cytokines secretion. This promoted the activation and recruitment of T cells into the TME and resulted in cancer suppression [50].

Although the majority of tumor associated macrophages (TAM) have been shown to adopt an M2-like phenotype (which is classified as anti-inflammatory/pro-tumor), the remarkable functional plasticity of macrophages enables them to exhibit different activation states (spanning M1-like to M2-like) depending on the immunostimulatory cytokines within the TME [51]. TAM represent a major immune cell subset that populate the TME and are commonly associated with poor prognosis [52]. In most cancers, they are regarded as machinery that amplify tumor promoting factors driving angiogenesis, invasion, and metastasis. In addition, TAM are indispensable in creating an immunosuppressive microenvironment via secretion of anti-inflammatory cytokines such as TGF-β, IL-10 and prostaglandin-E2 (PGE2), and modifying the immune cell composition within the TME; increase in the recruitment of immunosuppressive cell types (e.g., Tregs) while inhibiting the anti-tumor functions of tumor infiltrating lymphocytes (TILs) and NK cells [53,54]. Recently, it was reported that TAM may be the predominant source of LPA production in the ascites of ovarian cancer patients via a consecutive action by platelet-activating factor acetylhydrolase (PAF-AH) and ATX [12]. The expression levels of both enzymes were found to be higher in ascites-derived TAM than ovarian cancer cells. Moreover, LPA receptors were also differentially expressed in ovarian cancer cells and TAM. With both tumor cells and TAM expressing similar levels of LPAR1 and LPAR2, LPAR3 was predominantly expressed in tumor cells, while LPAR5 and LPAR6 were selective for TAM [12]. What role these LPA receptors play in terms of TAM functions remains to be elucidated.

### 2.2. Natural Killer Cells Responses to LPA

NK cells function to detect and kill physiologically stressed cells such as virally infected cells or tumor cells [55]. To date, there are only two articles describing a role of LPA in regulating NK cell function. In 2003, Jin et al. first reported that IL-2 activated human NK cells expressed LPAR1-3 and were capable of migrating towards an LPA gradient in a pertussis toxin (PTX)-sensitive manner, suggesting the involvement of the heterotrimeric Gαi proteins. The authors also found that LPA induced intracellular Ca^2+^ mobilization and increased interferon gamma (IFNγ) secretion in activated NK cells, a proinflammatory cytokine critical for viral and tumor eradication [35].

Another study in 2009 described the additional role of LPA in inhibiting the cytolytic functions of IL-2 activated human NK cells. The authors demonstrated that LPA via a PTX-independent manner blocked the release of perforin by human NK cells and prevented the cytolysis of human A2058 melanoma cells and Burkitt’s lymphoma cells. Although human NK cells express LPAR1-3, it was LPAR2 that was involved in mediating the inhibitory effect of LPA on NK cell functions via enhanced cAMP levels and activation of protein kinase A (PKA). Moreover, treatment of NK cells with cholera toxin, which activates adenylyl cyclase via Gs protein inhibited the cytotoxicity of NK cells towards tumor cells, indicating that the cAMP pathway is critical for this function [56]. Therefore, these in vitro studies appear to suggest that LPA can recruit NK cells via Gi proteins and inhibit its cytolytic function via the Gs-cAMP mediated pathway by activating different LPAR. Considering that LPA can be secreted by several tumor cells, it would be interesting to investigate these functions more thoroughly in an in vivo cancer model.

### 2.3. Neutrophil Responses to LPA

The link between LPA and neutrophils has been studied in lung-related diseases where it was demonstrated that LPA increased IL-8, a major neutrophil chemoattractant, principally through LPAR 1 and 3 [57]. In vivo, it has been shown that intratracheal LPA administration to mice stimulates the expression of MIP-2, the murine counterpart of IL-8, and neutrophil influx [58]. Similar findings were reported in guinea pigs, where LPA stimulated both eosinophil and neutrophil infiltration into the pulmonary airways [59]. However, in a mouse model of renal injury, a decrease in neutrophil influx following LPA treatment was documented [60]. In pneumonia patients, LPA exerts greater neutrophil chemoattraction compared to control subjects, and neutrophils have been reported to express LPAR1 [61]. Interestingly, it was demonstrated that epithelial ovarian carcinoma cells exposed to LPA and IL-8 were more invasive [62]. In cancer, numerous studies showed that neutrophils are involved in both pro- and anti-tumor processes. Studies from mice and humans support the evidence that these roles may be strongly related to the tumor stage [63]. Among the large heterogeneity of neutrophils and their diverse roles in tumorigenesis [63,64], IL-8 has been described to attract neutrophils into the TME and promotes tumor progression in several cancer types [65]. Tumor associated neutrophils have been associated with poor clinical outcome in most studies of human cancers [63]. Furthermore, due to its ability to display several pro-tumoral functions, neutrophils could be targeted for immunotherapy [63]. In this sense, a bigger picture of the interactions mediated by neutrophils is needed. Exploring the link between LPA and neutrophil subsets in tumorigenesis, for example by the ability of LPA to stimulate IL-8 and induce a neutrophil influx in the TME, would be of great interest to expand our current understanding and/or develop new anti-tumor approaches.

### 2.4. Eosinophil Leukocyte Regulation by LPA

In an in vitro model of allergy, it was shown that the eosinophil granule protein, eosinophil peroxidase, increases the expression of LPAR1-3 in the human cholinergic neuroblastoma cell line IMR-32, and that LPA can promote eosinophil recruitment by inducing the transcriptional over-expression of CCL26, an eosinophil chemoattractant [66]. LPA was also demonstrated to stimulate the infiltration of eosinophils in guinea pigs [59]. In a lung inflammation model induced by the parasite *Schistosoma mansoni*, the influx of eosinophils was attributed to LPAR2 [67]. In human eosinophils, LPA was reported as an inducer of chemotaxis and reactive oxygen species generation [28]. However, in allergic subjects, although LPA was detected in bronchoalveolar fluid, it did not appear to be a dominant chemoattractant for eosinophils [68]. Eosinophils are present in the TME of numerous human tumor types. They have been correlated with a good outcome in most cancers [69], except for cervical cancer [70,71], Hodgkin’s lymphoma [72,73], and lymphoma [74]. Taken together, investigating the ability of LPA in challenging an influx of eosinophils as a cancer therapeutic target would be relevant.

### 2.5. Modulation of Basophils by LPA

There have been limited studies investigating the relationship between LPA and basophils as well as basophils and cancer. One study showed that LPA stimulates the release of histamine by basophils, resulting in wheals and itch [75]. In parallel, early studies showed that the peripheral number of basophils in blood and degranulation were increased in bronchial carcinoma [76]. A recent study using Foxp3DTR mice depleted in Treg cells has shown that basophils induced the recruitment of CD8^+^ T cells into the tumor via the production of CCL3 and CCL4. Moreover, they demonstrated that basophils were essential for melanoma tumor rejection [77]. In contrast, in a mouse model of pancreatic ductal adenocarcinoma, it has been reported that basophil-deficient mice were protected from full tumor development compared to wild type mice [78]. Although more studies would be needed to determine how LPA or LPAR might affect basophil immune regulation, these few indications tend to demonstrate that basophils act as full players in tumorigenesis.

### 2.6. Mast Cells and LPA

LPA was reported to induce the proliferation and differentiation of mast cells (MC) by accelerating the acquisition of MC granules and increase the expression of Kit [31]. LPA was also reported to induce the production of pro-inflammatory cytokines via an LPAR2-mediated pathway requiring IL-4 priming and MAPK signaling [79]. LPA induced histamine release from MC [80], and consequently, plasma exudation in the skin [81]. More recently, LPAR5 has been identified as the dominant LPA receptor in human MC [82]. In parallel, a subpopulation of MC has been reported to express ATX in the human gastrointestinal tract [83]. In the context of atherosclerosis, the activation of MC by LPA enhanced the microvascular leakage in vivo, which could affect plaque stability. Regarding cancer, the position of MC remains poorly understood [84].

### 2.7. Dendritic Cells and LPA

Monocytes recruited to the site of inflammation are able to differentiate into DC depending on the environmental conditions [85]. In vitro, LPA was reported to affect monocyte-to-DC differentiation by blocking the expression of the surface molecule CD1a. However, this induced an impaired phenotype characterized by a different capability to drive naïve T cell polarization [86]. Another group demonstrated that LPA slightly enhanced the ability of immature DC to stimulate T cell proliferation and attenuated the ability of lipopolysaccharide to enhance IL-6 production from DC [87]. LPA exerts a different effect on immature and mature DC. On immature DC, LPA induces Ca^2+^ mobilization, actin polymerization, and chemotaxis [34]. Chemotaxis was shown to be mediated by LPAR3 in response to unsaturated LPA species [32]. Whereas on maturing DC, LPA reduces the production of IL-12 and TNFα and increases IL-10 [34], IL-6 and IL-8 [88]. LPA-induced IL-6 and IL-8 production was demonstrated to be regulated via PKC and Rho [88]. In a murine model of allergy, LPA was identified as a suppressor of DC activation through LPAR2 [89]. Besides LPA receptors, Leslie et al. [90] reported that LPA via PPARγ differentially regulates the development of group 1 and group 2 (CD1d) DC. Specifically, LPA-PPARγ axis reduced the transcription and cell surface expression of group 1-specific CD1 markers, and conversely increased CD1d, involved in lipid antigen presentation by DC to T cells. The various effects of LPA in modulating innate immunity are summarized in Table 1.

## 3. ATX-LPA-LPAR Signaling Axis in Regulating Adaptive Immunity

The adaptive immune system, which consists of T and B lymphocytes, is an important line of defense against cancer. T cells are the second immune cell type, after TAM, found in the TME. Activation of T cells is triggered when innate immune antigen presenting cells (APC) present tumor-associated Ag peptides in association with a major histocompatibility complex receptor that is recognized by and engages the T cell antigen receptor (TCR) and co-stimulatory molecules. This process leads to T cell proliferation, cytokine secretion, and recruitment of other effector cells. In turn, activated Th1 CD4^+^ T cells activate CD8^+^ T cells and activated Th2 CD4^+^ T cells stimulate B cells. Importantly, this mechanism is also negatively regulated by the expression of inhibitory molecules, such as PD-1 and CTLA-4, at the T cell membrane, via evolved physiological process that exist to normally terminate adaptive immune responses. Cancer cells can take advantage of this feature to suppress T cell cytotoxic functions by stimulating these inhibitory molecules prematurely. We report in this section, the effects of LPA on T and B lymphocytes in cancer.

### 3.1. B Lymphocyte Responses to LPA

The first evidence of an LPA effect on B lymphocytes was documented in 1998. Using Epstein Barr Virus (EBV)-immortalized human B cells as a study model, the authors demonstrated that LPA induced intracellular Ca^2+^ mobilization, MAPK activation, B lymphoblasts proliferation, and immunoglobulin (Ig) production [25]. In 2010, Nam et al. extended these studies to include mature and immature B cell lines, as well as freshly isolated mouse B cells and found that the effect of LPA on these cells varies significantly. Specifically, LPA increased intracellular Ca^2+^ levels via the phospholipase C (PLC)-dependent pathway and enhanced thapsigargin-induced store-operated Ca^2+^ entry (SOCE) only in mature Bal-17 B cells but not in immature WEHI-231 B cells. Interestingly, LPA had minimal effect on PLC-dependent Ca^2+^ mobilization in freshly isolated mouse splenic B cells (which consist of mature B cells) and bone marrow B cells (a mixed population of pro-, pre- and immature B cells). However, LPA was still capable of augmenting thapsigargin-induced SOCE, with a higher magnitude observed in bone marrow B cells compared to splenic B cells. Thus, it seems that LPA can induce different calcium responses based on the maturation status of B cells [93].

Isolated human B cells were found to express LPAR2 [24] whereas primary murine splenic B cells and the A20 B cell line were found to express LPAR2 and LPAR5 [24,94]. It remains to be determined if primary human B cells also express the LPAR5 since the study was conducted prior to the discovery of the receptor. Interestingly, we found that the newly discovered LPAR5 acts as an inhibitory receptor that blocks antigen-induced B cell receptor (BCR) signaling both in isolated murine splenic B cells and the A20 B cell line. BCR signaling is essential for the survival, development, proliferation, and antigen-driven priming of B cells into antibody-secreting cells. We demonstrated that in the presence of LPA, antigen induced Ca^2+^ mobilization and expression of CD69 and CD86 activation markers were all diminished in B cells. This inhibitory action of LPAR5 appears to be mediated by the Gα12/13-Arhgef1 pathway. Furthermore, *Lpar5*^−*/*−^ mice produced higher amounts of antigen-specific IgM compared to wild type littermates four days after immunization with NP-Ficoll, suggesting that LPAR5 may have a role in regulating humoral immune response [94].

While the majority of research on cancer immunity focuses on T cells, B cells have been found to be present in 25% of breast cancer cases and account for ~40% of the TILs population [95,96,97,98]. Moreover, infiltrating CD20^+^ B cells have also been reported in ~40% of high grade serous ovarian cancer [99]. Their anti-tumor role may consist of serving as APCs, producing tumor-reactive antibodies, and secretion of cytokines and chemokines to regulate T cell responses [98]. Not much is known in terms of the function of ATX-LPA-LPAR signaling in tumor infiltrating B lymphocytes (TIBLs) except an earlier study by Yang et al. in 1999 where they discovered that non-small-cell lung cancer biopsies had a significant infiltration of CD20^+^ TIBLs, which showed intense staining of ATX compared to adjacent normal bronchial epithelium [100]. In line with this, Lin et al. recently reported that B cells are a major source of ATX in human and mouse colon and contribute to the severity of inflammation in chronic colitis [101]. Thus, the significance of high ATX expression in B cells in the context of cancer remains unknown and warrants further investigation.

### 3.2. T Lymphocyte Responses to LPA

The regulatory role of LPA on T cell function is well established but is complex and confounded by several limitations. First, qPCR data, in several reports, confirm the simultaneous expression of multiple LPAR in T cells. Second, the expression of LPAR in T cells changes in response to their activation state. For example, naïve CD4^+^ T cells predominantly express LPAR2 over LPAR1 and exposure of these cells to LPA results in inhibition of IL-2 secretion. However, after activation with the mitogen phytohemagglutinin (PHA), LPAR1 expression increases instead, and LPA enhanced TCR activated IL-2 secretion [102]. The third confounder is the lack of availability of high-specificity agonists and antagonists to LPAR. In this context, the seminal work by the Goetzl group using LPAR1 and LPAR2 specific activation monoclonal antibodies (MAb) raised to a peptide composed of residues of 9-27 LPAR has been instrumental in validating the pharmacological results obtained with LPA in PHA-stimulated CD4^+^ T cells [103]. They showed that anti-LPAR2 MAb stimulation inhibits, whereas anti-LPAR1 MAb stimulation enhances, TCR-activated IL-2 production in human CD4^+^ T cells and in CD3^+^ PMA activated Jurkat T cells. Thus, the shift in LPAR2 predominance to LPAR1 and LPAR2 codominance represents an important aspect of LPAR plasticity caused by mitogen activation of human CD4^+^ T cells. Another instance of LPAR mediating different biological responses in T cells was demonstrated by Zheng and colleagues. The authors examined the transmigration of Jurkat T cells with forced expression of either LPAR1 or LPAR2, across polycarbonate filters coated with basement membrane-like Matrigel. Treatment of LPA (10^−9^–10^−6^ M) or the anti-LPAR2 MAb (30–300 ng/mL) stimulated the transmigration of LPAR2 Jurkat transfectants with a concomitant increase in matrix metalloproteinase secretion and the production of the migratory chemokine RANTES. In contrast, neither LPA nor the anti-LPAR1 MAb induced a migratory response in LPAR1 Jurkat transfectants [104]. Taken together, these studies convey two important points; first, LPAR can be differentially expressed based on the activation state of T cells and secondly, these LPAR may exert different or opposing biological functions. Hence, the response to LPA may represent the summation of the varying actions mediated by the different LPAR subtypes being expressed in either naïve or activated T cells. This observation might be relevant to the migratory behavior of activated T cells that upregulate LPAR1 expression causing inhibition of T cell homing and migration when exposed to high levels of LPA in the TME. Conversely, LPAR2 might play an important role in the penetration of naïve T cells into target tissues. Thus, the mechanism that regulates the dynamic interplay between LPAR expression and how it affects the behavior of T cells in the TME remains unknown and warrants further investigation.

LPA has also been documented in lymphocyte homing to secondary lymphoid organs. In particular, high amounts of ATX are expressed by cuboidal high endothelial venules (HEV), which are specialized blood vessels found in lymphoid tissues that function as portals for lymphocyte entry to lymph nodes [105]. A subsequent report noted that LPAR4 and LPAR6 are highly expressed in HEV cells and selective individual knockout of these receptors in mice leads to the accumulation of lymphocytes in the endothelial cell layer and unable to enter the lymph node [106]. The mechanism of LPA-dependent homing of lymphocytes into sites of metastasis is not clear, although the involvement of LPAR1, LPAR5 in the seeding of B16 melanoma-derived lung metastasis has been demonstrated using knockout mice for these LPAR subtypes [107]. It has been demonstrated that some tumor infiltrating blood vessels in melanoma, breast and ovarian carcinoma biopsy specimen share many of the properties of HEV cells. These tumor-associated HEVs function as portals for the entry of naïve, central memory, and activated effector memory T cells into the TME to facilitate tumor destruction [108]. In a study with 225 melanoma patients, the density of HEV-like cells in the tumor correlated with reduced tumor size, expression of Th1 marker genes and accumulation of dendritic cells [109,110]. Although the expression of the peripheral node Addressin protein marker in primary breast intratumoral vessels, which is considered a marker of HEV, was correlated with longer disease-free survival; extratumoral HEV at the invasion margin in colorectal cancer correlated with a poor prognosis [108]. The HEV-like properties of the endothelium in tumor-induced vessels remains poorly understood and somewhat controversial, hence it requires more research [111,112].

The concentrations of LPA active in modulating lymphocyte responses are remarkably low with maximal efficiency in the 10^−10^ to 10^−8^ M range characterized with a bell shape dose-response curve dropping off above 1 µM [102,104]. This is three orders of magnitude lower than the critical micellar concentration values of LPA which are 1.850, 0.540, 0.082, and 0.346 mM, respectively, when the acyl group is myristoyl, palmitoyl, stearoyl, and oleoyl [113]. Although no direct evidence for the actual LPA concentration in the TME has been reported yet, the subnanomolar in vitro potency of LPA in T cells suggests that the 10–100 nM plasma concentration of LPA [11,114] is sufficient to evoke LPAR activation of immune cells. Indeed, nanomolar to subnanomolar concentrations of LPA have been demonstrated to protect CD4^+^CD8^+^3low Tsup-1 human T cell line from apoptosis induced by a combination of antibodies to Fas, CD2, or CD3 plus CD28 [103]. The signaling mechanism behind the antiapoptotic effect of LPA was mediated via a reduction in Bax and did not affect Bcl2 or Bcl-xL levels in these cells. Moreover, treatment with antisense RNA to LPAR1 and LPAR2 completely abolished the anti-apoptotic effect of LPA, suggesting that these two LPAR are critical in protecting T cells from apoptosis. In the Jurkat T cell line, An and colleagues [115] showed by transfecting LPAR1 or LPAR2 together with a SRE-driven luciferase reporter gene that LPAR2 robustly (20-fold over control) but LPAR1 to a lesser extent (5-fold over control) induced LPA-dependent luciferase expression. LPAR2 mediated reporter gene expression was partially sensitive to PTX and the C3 toxin indicating Gi and Rho GTPase dependent signals. The Jurkat cell subline used for transfection by these authors showed significant specific binding of [3H-LPA] along with weak Ca^2+^ mobilization upon LPA treatment but only trace expression of LPAR1 and LPAR2 based on semiquantitative PCR method. These authors suggested that the responses in vector transfected cells could have been due to a yet unidentified LPAR at the time. It is important to note that these studies predated the discovery of the non-EDG LPAR members LPAR4, 5 and 6.

**Figure 2:**
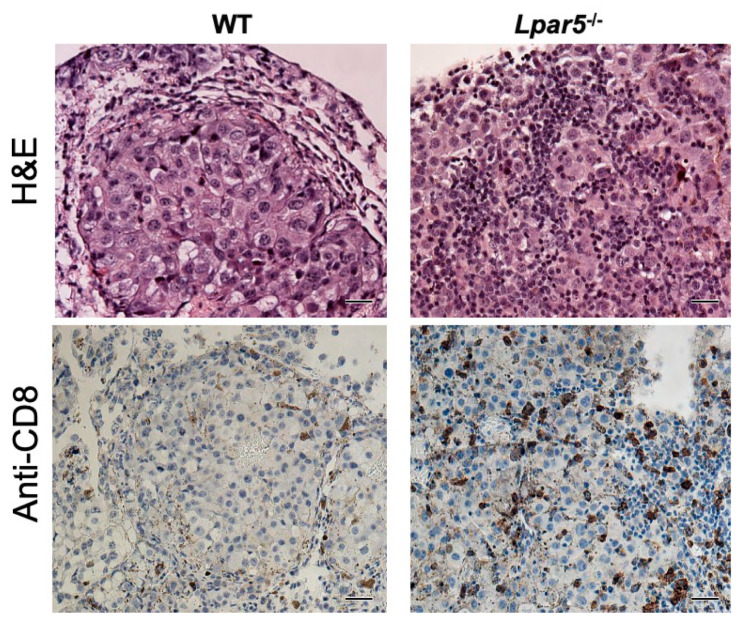
Increased infiltration of CD8^+^ T cells in lungs of *Lpar5*^−/−^ mice compared to wild type mice (WT). In the few *Lpar5*^−^^/−^ mice (*n* = 3 out of 12 mice) that had low numbers of metastatic tumors, higher incidence of lymphocyte infiltration was observed and positively stained for CD8. No or lesser lymphocyte infiltration was seen in wild type littermates. Scale bar (50 µm), 200× magnification.

It was only later that we discovered that LPAR5 is abundantly expressed in murine and human CD8^+^ T cells and functions as an inhibitory receptor that blocks the anti-tumor actions of effector CD8^+^ T cells. Specifically, activation of LPAR5 by LPA blocked antigen-induced activation of TCR, intracellular Ca^2+^ mobilization, ERK signaling, and prevented CD8^+^ T cells from proliferating [43,116]. In addition, stimulation of CD8^+^ T cells with a metabolically stabilized analog of LPA, octadecyl thiophosphate (OTP), impaired granule exocytosis resulting in a decreased in vitro tumor cell killing function by both mouse and human CD8^+^ T cells [43]. These in vitro data were further supported by in vivo observations that adoptive transfer of CD8^+^ T cells isolated from *Lpar5*^−*/*−^ mice were more effective at reducing the growth rate of both EG7 lymphoma and B16F10 melanoma tumors compared to wild type CD8^+^ T cells [43,116]. In a separate study, the incidence of B16F10 melanoma-derived lung metastases were found to be reduced by 85% in *Lpar5*^−*/*−^ mice [107]. In the few mice that presented lung metastasis, an increase infiltration of CD8^+^ T cells were observed compared to wild type littermates (Figure 2). Taken together, these studies suggest that inhibition of LPAR5 in distinct immune cell types such as CD8^+^ T cells may boost anti-tumor activity and impede metastasis.

Interestingly, a recent study [41] found that ATX secreted by melanoma cells acts as a chemorepellent to block migration of TILs into the tumor sites. In particular, the authors showed that conditioned media derived from ATX-expressing melanoma cells suppressed the basal migration of both TILs derived from melanoma patients and peripheral CD8^+^ T cells isolated from healthy donors. No effect was observed when conditioned media from ATX knockdown melanoma cells was used. When these experiments were performed in the presence of ATX inhibitors (e.g., PF-8380 or IOA-289), conditioned media derived from ATX-expressing tumor cells failed to inhibit the migration of T cells, suggesting that the chemorepellent effect of ATX was due to the production of LPA. Indeed, treatment of LPA not only suppressed the spontaneous migration of TILs and CD8^+^ T cells, but also inhibited their migration towards the chemokine CXCL10. These in vitro findings were confirmed in vivo using a mouse vaccination tumor model where they demonstrated that melanoma tumors with high ATX expression had less infiltrating CD8^+^ T cells compared to melanoma tumors with low ATX expression. Furthermore, single-cell RNA seq analysis of 32 clinical melanoma samples showed an inverse correlation of ATX expression in tumor cells with intratumoral CD8^+^ T cells accumulation. This finding from human patients agrees with our findings using *Enpp2^+/^*^−^ mice that showed enhanced tumor cell killing compared to wild type mice [43]. The chemorepellent effect of ATX/LPA was proposed to be mediated in part by activation of LPAR6 on TILs. Specifically, TILs derived from three melanoma patients predominantly expressed LPAR6 with low expression of LPAR2, whereas peripheral CD8^+^ T cells expressed LPAR6 > LPAR2 > LPAR5 > LPAR4. A combination of factors such as ex vivo culture expansion or in situ immunoediting in the TME may in part account for the loss of LPAR5 and LPAR4 expression in patient derived TILs compared to naïve CD8^+^ T cells [41]. In line with this study, tumor associated T cells isolated from the ascites of ovarian cancer patients also showed higher expression of LPAR5 and LPAR6, whereas LPAR1-4 expression was comparable to ovarian cancer cells [12].

**Figure 3:**
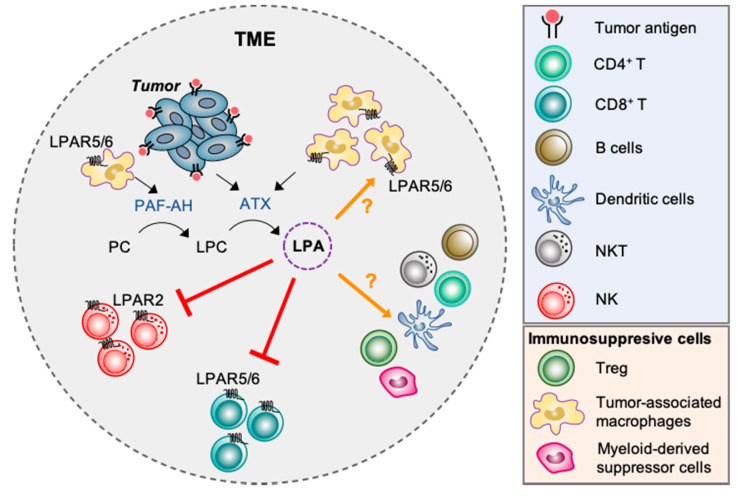
Potential role of ATX-LPA-LPAR signaling axis in the regulation of cancer immunity. Upregulation of LPA in the tumor microenvironment (TME) may serve as an inhibitory mechanism that suppress anti-tumor immunity via modulating the function of different immune cell types. For example, LPA may suppress the cytolytic actions of natural killer (NK) cells against tumor cells via LPAR2; block antigen-induced CD8^+^ T cell activation, proliferation and tumor-cell killing via LPAR5 receptor; and inhibit migration of CD8^+^ T cell into the tumor via LPAR6. Furthermore, the recruitment of TAM into the TME may serve as another source of LPA via the actions of PAF-AH and ATX. How LPA regulates TAM, CD4^+^ T cells, B cells, NKT cells, T reg and myeloid-derived suppressor cells in the TME remains to be determined.

Taken together these studies underscore a prominent role of ATX-LPA-LPAR signaling axis in the regulation of cancer immunity while at the same time highlight a critical need to expand our understanding in this area of research. In particular, many different types of immune cells can infiltrate the TME and the effects of LPA on these cells remain largely unexplored (Figure 3).

## 4. Conclusions

Since the clinical success of ipilimumab, a checkpoint inhibitor targeting CTLA-4 in 2011, at least 20 other immunotherapies have been approved by the U.S. Food and Drug Administration (FDA) to treat cancers of the skin, lung, bladder, kidney, stomach, liver, prostate, breast, multiple myeloma, leukemia, and Hodgkin’s diseases [117]. Although immunotherapy is highly effective, only a small fraction of patients within a large cohort respond favorably to the treatment. A recent 2018 study estimated that at least 44% of cancer patients are eligible for current checkpoint inhibitor drugs, with 13% of patients estimated to respond to these therapies, a modest improvement from the 0.14% response rate in 2011 [118]. Hence, understanding the mechanisms that regulate immune response to cancer is warranted in order to develop treatment strategies that benefit a larger percentage of patients.

The emerging data on the role of LPA signaling axis in regulating cancer immunity demonstrates the value of this target in cancer therapy. As an example, inhibition of LPAR5 in distinct cell types, such as CD8^+^ T cells could help boost its immunosurveillance activity against cancer and impede metastasis. There is currently no FDA-approved LPA receptor antagonist for cancer therapy. A thorough review of the literature revealed three partially characterized LPAR5 antagonists: (TC-LPA5-4 [119], UA-02-085 [120], and AS2717638 [121]) in cell-based assays using human platelets, human MC, murine microglial cells, and in rat models of neuropathic pain, respectively. We obtained both TC-LPA5-4 and UA-02-085 and tested for LPAR5 antagonism in two independent assays: Ca^2+^ mobilization [122] and β-Arrestin recruitment. As shown in Table 2, UA-02-085 inhibited LPA-induced Ca^2+^ mobilization and β-Arrestin recruitment with an IC_50_ of 3 µM and 16.9 µM, respectively. Contrary to the findings published by Kozian et al. [119] we found that TC-LPA5-4 was inactive in both assays. The alkyl ether analogs of LPA, designated as alkyl glycerophosphate (AGP), are the most potent LPA species at activating LPAR5 [123,124,125,126] and PPARγ [127,128]. AGP is present in several tissues and biological fluids including tumor ascites [129], atherosclerotic plaques [130], aqueous humor of the eye [131], and in platelet rich plasma [132,133,134,135]. Thus, alkyl-LPA might have multiple actions in the TME as well as the cancer cells. Intriguingly, we found that both UA-02-085 and TC-LPA5-4 failed to inhibit AGP-induced Ca^2+^ mobilization (unpublished). We included as a positive control, Amgen35, a compound originally discovered by AMGEN investigators to be a LPAR2 antagonist with an IC_50_ of 0.8 µM [136]. The AMGEN publication predated the discovery of the LPAR4-6. When screened against these receptors, we found that Amgen35 at remarkably high concentrations inhibited LPAR5 in both assays, suggesting that the discrepancy observed with TC-LPA5-4 is unlikely due to the assays employed (Table 2). Amgen35 has also been reported to have off-target effects in vivo [136]. Additional selectivity screening at other LPAR and ATX revealed that UA-02-085 also inhibited LPAR4 (IC_50_ = 0.9 µM), and both TC-LPA5-4 and UA-02-085 inhibited ATX with an IC_50_ of 7.1 µM and 3 µM, respectively, casting doubt on their in vivo utility and specificity (Table 2). The other putative LPAR5 antagonist compound, AS2727638, reported by Astellas Pharma in Japan, has only been characterized at LPAR1-3 but not at the other LPAR with high homology of LPAR5 [121].

The relatively recent understanding that tumors avoid elimination by promoting inhibitory receptor signaling by tumor-specific CD8^+^ T cells has led to remarkable, but limited, success in the treatment of certain cancers through the use of checkpoint blockade therapies. To improve on this success, in the future we will need to consider combinations of checkpoint blockade therapies, each targeting a distinct suppressive pathway. In this regard, we consider that antagonism of LPAR5 signaling by tumor-specific CD8^+^ T cells may be an important addition to the arsenal of checkpoint inhibitors. Importantly, unlike the currently targeted CD8^+^ T cell inhibitory receptors, PD-1 and CTLA-4, which are expressed only after a CD8^+^ T cell has been activated, LPAR5 signaling would be expected to also lower the activation threshold of naïve tumor-specific CD8^+^ T cells displaying weak affinity for tumor antigens ultimately promoting a more robust anti-tumor CD8^+^ response.

## Figures and Tables

**Table 1:** Effects of lysophosphatidic acid (LPA) in Immune Cell Types of Innate Immunity.

Cell Type	Physiological Roles	Regulation through LPA in Pathological Context
Neutrophils	The most abundant leukocytes in the bloodstream. Considered as the first line of defense in the innate immune system through their ability to trap, capture, and phagocytose microorganisms	LPA induces neutrophil recruitment in vitro [58], in vivo [59] and in pneumonia patients [61], but decreases the recruitment of neutrophils in a model of renal injury [60]
Eosinophils	Associated with inflammatory responses, particularly caused by parasitic infection and allergic reaction	Eosinophil peroxidase increases the expression of LPAR1 and LPAR3 in human cholinergic neuroblastoma cells [66] LPA promotes eosinophil recruitment [66] and infiltration in guinea pigs [59] but not in allergic subjects [68]
Basophils	Involved in hypersensitivity, chronic inflammation and immune cell memory [91]	LPA stimulates histamine release from basophils [75]
Mast cells	Function principally as effectors of allergic inflammation	LPA induces histamine release from MC [80] and the proliferation and differentiation of MC through LPAR1,3 and PPARγ [31]
Macrophages	Maintain tissue homeostasis, role in controlling angiogenesis and metabolism [92] LPA differentiates monocytes into macrophages [44]	Pro-neoplastic actions of LPA: LPAR3 and LPAR2 are associated with macrophages infiltration in breast cancer [48] and colitis-associated cancer [49] TAM may be the predominant source of LPA production in the ascites of ovarian cancer patients [12] Anti-neoplastic actions of LPA: LPA via LPAR1 and LPAR3 polarizes macrophages into a M1 phenotype, which in turn activate T cells and recruit them into the TME [50]
Dendritic cells	Antigen-presenting cells capable of inducing naïve T cell activation and effector differentiation	LPA affects monocyte-to-DC differentiation but impairs antigen presentation by DC and alter DC phenotype [86] LPA induces different effects that are dependent on the activation state of DC: In immature DC, LPA induces calcium mobilization, actin polymerization, chemotaxis [34], and enhances the ability of DC to stimulate T cell proliferation [87] but suppresses DC activation through LPAR2 in vivo [89] In mature DC, LPA reduces IL-12 and TNFα and increases IL-10, IL-6 and IL-8 production [34,88]
NK cells	Detect and kill physiologically stressed cells [55]	Pro-neoplastic actions of LPA: It blocks the release of perforin by NK cells and prevents the cytolysis of human cancer cells in vitro, through LPAR2 [56] Anti-neoplastic actions of LPA: In vitro, LPA induces the chemotaxis of NK cells, intracellular calcium mobilization and enhances IFNγ secretion in activated NK cells [35], which in turn might augment elimination of neoplastic cells

**Table 2:** Characterization of Amgen35, UA-02-085 and TC-LPA-5 compounds at LPAR2, LPAR4, LPAR5, and ATX. UA-02-085 and Amgen35 blocked LPA-induced Ca^2+^ mobilization in LPAR5 expressing B103 cells and β-arrestin recruitment in LPAR5 expressing CHO-K1 cells. TC-LPA-5 had no effect on all LPAR tested. Both UA-02-085 and TC-LPA-5 inhibited ATX activity measured using the fluorescent FS-3 substrate assay.

ATX and LPAR Inhibition by Published LPAR Antagonists					
Compounds	ATX IC_50_	LPAR2 IC_50_	LPAR4 IC_50_	LPAR5 IC_50_ (Ca^2+^ Mobilization)	LPAR5 IC_50_ (β-arrestin)
Amgen35	NT	0.8 ± 0.5	NE	60.5 ± 10.1	99.2 ± 30.0
UA-02-085	3.0 ± 0.3	NE	0.9 ± 0.2	3.0 ± 0.3	16.9 ± 2.8
TC-LPA-5	7.1 ± 0.6	NE	NE	NE	NE

Values represent IC50 ± SD in µM. NT = Not tested; NE = No effect.
